# Different flames, different fates: A comparative study of surgical interventions and critical care burden in electrical vs. thermal burn injuries

**DOI:** 10.1007/s00508-025-02686-5

**Published:** 2026-01-05

**Authors:** V. Koenig, A. Christ, A. Resch, G. Ihra, M. Monai, J. Joestl, A. Fochtmann

**Affiliations:** 1 Division of Plastic, Aesthetic and Reconstructive Surgery, Medical University of Vienna, Waehringer Guertel 18–20, 1090 Vienna, Austria; 2 Division of General Anaesthesia and Intensive Care Medicine, Medical University of Vienna, Waehringer Guertel 18–20, 1090 Vienna, Austria; 3 Private Clinic: PD Dr. Julian Joestl, PhD, MSc., Spitalgasse 19, 1090 Vienna, Austria

**Keywords:** High-voltage electrical injury, Electrical trauma, Severe burn, Multisystem organ failure, Acute kidney injury, Mortality

## Abstract

**Background:**

High-voltage electrical injuries (HVEI) and thermal burns are both classified as severe burn trauma, yet their clinical impact differs markedly. The HVEIs cause deep tissue destruction and systemic complications that are often underestimated in severity by conventional burn indices such as TBSA (Total Body Surface Area) and ABSI (Abbreviated Burn Severity Index).

**Methods:**

A retrospective cohort analysis was performed on 1515 burn patients treated between 1994 and 2024. After exclusions 1489 patients were evaluated, including 92 with HVEI and 1397 with thermal burns. The demographics, burn size, ABSI, intensive care unit (ICU) stay and surgical procedures were analyzed using nonparametric tests.

**Results:**

Across the cohort, the mean number of operations was 3.56 (median 3). The HVEI patients required significantly more procedures than thermally injured patients (mean 5.01 vs. 3.49; *p* < 0.001). Burn size (*p* < 0.001), ABSI (*p* < 0.001) and ICU stay (*p* < 0.001) were all significantly associated with the number of operations. No sex-related differences were observed (*p* = 0.67).

**Conclusion:**

The HVEIs impose a disproportionately high surgical and intensive care burden compared to thermal burns. These findings highlight the limitations of current severity scores and emphasize the need for early recognition, repeated surgical management and tailored interdisciplinary care in patients with electrical trauma.

## Introduction

Burn injuries, whether caused by flame, scalding or electricity, represent one of the most resource-intensive forms of trauma, often requiring complex surgical management and prolonged critical care [1–4]. Among them, high-voltage electrical injuries (HVEIs) stand out for their unique pathophysiology and devastating impact on multiple tissue layers and organ systems [5–14]. While both thermal and electrical burns may present with similar total body surface area (TBSA) involvement, HVEIs frequently cause disproportionately severe internal injuries due to the deep conduction of current, leading to profound muscle necrosis, vascular damage and systemic complications [15–17].

Despite these fundamental differences in injury mechanism and tissue involvement, clinical guidelines and triage tools often rely on TBSA and depth alone, without adequately accounting for the distinct challenges posed by electrical trauma [1, 18]. Thermal burns typically result in surface-level tissue destruction, while electrical injuries carry a higher risk of compartment syndrome, amputations and secondary organ failure, including cardiac, renal, and hepatic dysfunction [19–22]. These differences may translate into varying needs for intensive care, the number and type of surgical interventions and the overall resource burden per patient.

In recent years, management has transitioned toward individualized, hemodynamic-guided management. This approach emphasizes the use of balanced crystalloids, albumin and high-dose vitamin C in minimal effective volumes, with treatment decisions supported by advanced monitoring techniques such as pulse index contour cardiac output (PiCCO), bedside ultrasound and echocardiographic assessment. These developments reflect a shift toward precision critical care, aiming to optimize fluid balance, tissue perfusion and systemic stability in severely burned patients [1, 6, 18].

High-voltage electrical injuries (HVEI) are characterized by a distinct pathophysiology that helps explain the high rates of amputations and systemic failure observed in these patients [23]. Unlike thermal burns, electrical current travels along the path of least resistance, preferentially through nerves, vessels and muscle, resulting in profound deep-tissue necrosis that is often underestimated on initial inspection [1, 18]. Progressive myonecrosis increases compartmental pressures and compromises distal perfusion, frequently necessitating early fasciotomy and, in severe cases, major limb amputation. Moreover, extensive muscle destruction leads to massive release of myoglobin and creatine kinase, which, together with renal hypoperfusion, contributes to pigment-induced acute kidney injury [5]. This cascade of deep tissue necrosis, ischemia-reperfusion injury and myoglobin-mediated renal toxicity, may subsequently trigger multiorgan failure [18].

Understanding the comparative burden of surgical and intensive care interventions between these two injury types is essential for optimizing triage, resource allocation and treatment pathways. To date, few studies have systematically compared the treatment trajectory of HVEI patients with that of thermal burn patients. This retrospective matched case-control study aims to fill this gap by comparing the critical care and surgical burden in patients with HVEI to those with severe thermal burns. By matching cohorts based on age and TBSA, we aim to isolate the impact of the injury mechanism on treatment intensity and outcomes, providing insights into whether electrical trauma should be considered a distinct clinical entity with different care needs and prognostic implications.

## Material and methods

After receiving approval from the institutional ethics committee (reference number: 1384/2023), we performed a retrospective cohort analysis of all burn patients treated at our burn center between January 1994 and December 2024. Patient data were retrieved from the hospital’s electronic medical record system, anonymized and systematically reviewed.

The study population was divided into two groups:

1. Individuals who sustained high-voltage electrical injuries (HVEI) and were admitted between 1994 and 2024 and
2. Patients presenting with thermal burns, treated from 2000 to 2024.

Inclusion criteria comprised all patients with confirmed high-voltage trauma (≥ 1000 V), including injuries related to train surfing or occupational incidents. No restrictions were placed on age or sex. Patients with low-voltage injuries or incomplete medical records were excluded from analysis.

In total, 1515 patients met the inclusion criteria: 92 patients with HVEI and 1427 with thermal burn injuries. For each case, the following clinical parameters were assessed: sex, age, percentage of total body surface area burned (TBSA), abbreviated burn severity index (ABSI), duration of ICU stay, requirement for dialysis and in-hospital mortality.

In cases of electrical injury, the suspected current path and associated internal damage were additionally documented. Acute kidney injury (AKI) was identified based on clinical assessment and documentation in the patient file. Dialysis was considered present if continuous renal replacement therapy or intermittent hemodialysis was administered during the ICU stay. In-hospital mortality was defined as death occurring during the initial hospitalization period.

All patients were managed following standardized institutional burn care protocols. Core measures included structured fluid resuscitation guided by urine output, hematocrit and lactate levels, alongside daily wound care, early excision of necrotic tissue and timely surgical coverage. Therapeutic strategies encompassed the use of polyurethane dressings, negative pressure wound therapy (VAC), split-thickness skin grafting and flap reconstruction, with microsurgical free flaps applied in selected cases. Patients with electrical trauma and suspected compartment syndrome underwent immediate fasciotomy.

Continuous variables were assessed for normality. Normally distributed variables are reported as mean ± standard deviation (SD), while non-normally distributed variables are presented as median (interquartile range, IQR). Categorical variables are reported as counts and percentages. Group comparisons between HVEI and thermal burn patients were performed using the Mann-Whitney U test for continuous variables and the χ^2^-test for categorical variables. Correlations were assessed using Spearman rank correlation. A *p*-value < 0.05 was considered statistically significant.

## Results

**Fig. 1:**
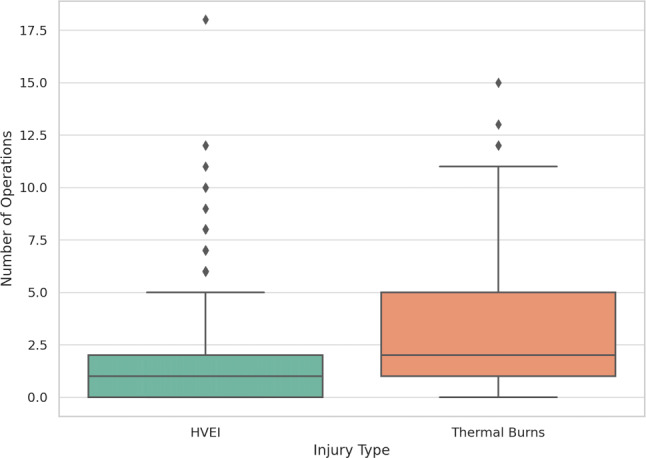
Graph showing the number of operations by injury type. *HVEI* high-voltage electrical injuries

In this cohort of 1515 burn patients, comprising 92 patients with HVEI and 1423 with thermal burns, the number of surgical procedures per patient was analyzed to quantify treatment burden. After excluding entries with missing or invalid data, 1489 patients were included in the final statistical evaluation. Across the entire cohort, the mean number of operations per patient was 3.56, with a median of 3. The number of surgical procedures ranged widely, from 0 to more than 20 in individual cases, reflecting the broad variability in clinical severity and surgical needs (Fig. 1).

**Fig. 2:**
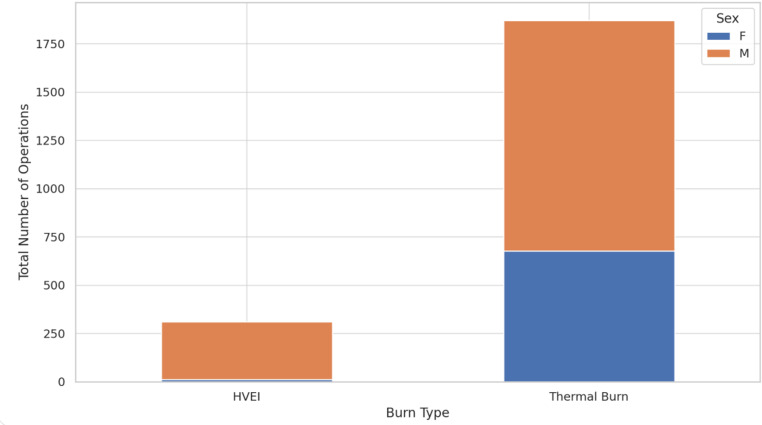
Number of patients operated by sex and burn type

When stratified by sex, 956 of the analyzed patients were male (64.2%) and 533 were female (35.8%). Male patients had a mean of 3.59 operations (median: 3), while female patients had a mean of 3.50 (median: 3) (Fig. 2). The difference between sexes was not statistically significant (*p* = 0.67, Mann-Whitney U test), suggesting that the overall surgical burden was comparable between men and women, despite potential differences in burn characteristics.

However, when comparing the two injury mechanisms, a significant difference emerged. Patients with HVEI underwent a mean of 5.01 surgical procedures (median: 4), whereas those with thermal burns had a mean of 3.49 operations (median: 3). This difference was statistically significant (*p* < 0.001, Mann-Whitney U test), indicating that HVEI patients required more surgical interventions. This finding is consistent with the deeper and often underestimated tissue damage caused by electrical current, frequently necessitating repeated debridement, fasciotomy and reconstructive procedures. Furthermore, correlation analyses demonstrated that the number of operations was significantly associated with several indicators of clinical severity.

**Fig. 3:**
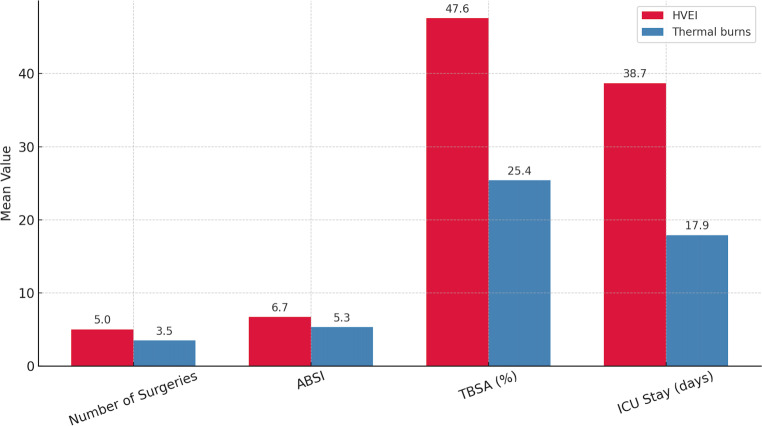
Comparison of HVEI and burn patients: surgeries, abbreviated burn severity index (ABSI), total burned body surface area (TBSA) and intensive care unit (ICU) stay

The total burned body surface area (TBSA) correlated positively with the number of operations (*p* < 0.001) as did the abbreviated burn severity index (ABSI) (*p* < 0.001). Notably, ICU stay showed the strongest correlation (*p* < 0.001) suggesting that prolonged intensive care treatment is closely linked to a higher number of surgical procedures (Fig. 3).

In summary, while sex did not significantly influence the number of operations, patients with HVEIs required more surgeries compared to those with thermal burns. The number of operations increased proportionally with burn size, ABSI score and ICU length of stay.

In the HVEI cohort (*n* = 92) limb loss was common, with amputations required in 32 patients (34.8%). Major limb loss occurred in 14 patients (15.2%), whereas minor amputations, predominantly involving toes and fingers, were noted in 18 patients (19.6%). In contrast, amputations were considerably less frequent among thermally injured patients (6%; *p* < 0.001) and major amputations were rare (1.9%; *p* < 0.01).

Mortality among male and female HVEI patients was also higher (10–15%) compared with thermally burned individuals (6.8%; *p* < 0.05).

These findings reflect the extensive and complex surgical demands in high-voltage trauma and underline the importance of tailored surgical strategies and interdisciplinary care (Table 1).

**Table 1:** Comparison of HVEI vs. thermal burns

Variable	HVEI—Mean ± SD	HVEI—Median (IQR)	Thermal burns—Mean ± SD	Thermal burns—Median (IQR)
TBSA (%)	25.64 ± 23.25	18 (5–45)	24.22 ± 21.97	16 (8–30)
ICU stay (days)	23.57 ± 33.86	14 (4–32)	18.60 ± 28.84	8 (1–23)
ABSI	5.70 ± 2.16	5 (4–7)	6.97 ± 2.95	6 (5–9)
Operations (n)	5.01 ± 3.35	4 (1–5)	3.49 ± 1.71	3 (0–2)

*TBSA Total body surface area, ICU intensive care unit, ABSI abbreviated burn severity index*

## Discussion

Our findings underscore that although HVEIs and thermal burns are often grouped together under the umbrella term of “severe burn trauma”, their clinical trajectories diverge significantly. In our cohort, HVEI patients underwent a mean of 5.01 surgical procedures compared to 3.49 in thermally burned patients. This difference remained significant even after accounting for TBSA and ABSI, suggesting that HVEI represents not merely a subtype of burns but a multisystem injury with a distinct surgical and critical care burden.

When directly compared with thermally injured patients, HVEI patients present with a more complex trajectory. Stockly et al. analyzed 1147 burn survivors and found that individuals with electrical injuries reported significantly worse physical health and were nearly half as likely to be employed 2 years after injury compared to survivors of fire and flame burns [16]. This is a pivotal finding: despite both groups being “severe burn patients,” electrical trauma results in persistent disability and impaired reintegration into working life. Our findings are consistent with these results: the higher acute surgical and ICU burden we observed in HVEI patients likely contributes directly to their poorer long-term physical outcomes. Importantly, it highlights that merging electrical and thermal burns into a single prognostic group may obscure the distinct challenges associated with electrical trauma.

Koenig et al. reported in their 30-year review that train-surfing injuries required more operations and longer ICU stays than occupational HVEI, highlighting the heterogeneity even within electrical trauma [1, 5]. Our results complement these data by showing that beyond intra-HVEI comparisons, electrical trauma also places a higher surgical demand than thermal burns of comparable extent. Similarly, Koenig et al. demonstrated that vertical current pathways were strongly associated with cardiac and renal complications [5]. In our analysis, ICU stay correlated most strongly with number of operations, reflecting the systemic impact of HVEI that extends beyond cutaneous injury.

Other international series confirm that HVEI entails greater morbidity than thermal burns. Korkiamäki et al. described train climbers requiring on average five operations and prolonged hospital stays, paralleling our observation of higher surgical intensity [2]. Ding et al. likewise reported more amputations, complications and longer hospitalization in HVEI patients compared to low-voltage and thermal cohorts [24]. Shih et al. in a large review concluded that HVEIs consistently carry higher morbidity and mortality than low-voltage or thermal burns [19]. Our study reinforces these findings with direct intra-cohort evidence, showing that even within a burn population, the injury mechanism profoundly shapes treatment needs.

At the same time similarities remain: both electrical and thermal burns share risks for infection, sepsis and acute kidney injury. Kim et al. demonstrated that septic burn patients frequently develop AKI, independent of the burn mechanism [25]. Our data likewise showed that TBSA and ABSI correlated with the number of operations in both groups, indicating overlapping determinants of surgical demands. Mariano et al. further showed that burn patients with AKI can preserve long-term renal function after continuous renal replacement therapy (CRRT) but remain predisposed to chronic comorbidities [4]. This underscores the need for close long-term follow-up in both patient populations.

In conclusion, our results and the literature converge on a key point: while both HVEI and thermal burns are life-threatening and resource-intensive injuries, HVEI must be conceptualized as a distinct multisystem trauma. The deeper tissue involvement and systemic sequelae translate into higher operative requirements, prolonged ICU stays and worse long-term outcomes than thermal burns of comparable severity. Future triage and prognostic tools should reflect these differences, ensuring that electrical trauma is not simply subsumed under general burn care but recognized for its unique clinical challenges.

## Conclusion

This study demonstrates that high-voltage electrical injuries (HVEI) constitute a complex trauma entity with a disproportionately high surgical and intensive care burden. In our cohort, patients with HVEI underwent significantly more operations than other burn patients and the number of surgical procedures strongly correlated with TBSA, ABSI and particularly ICU length of stay. These findings reflect the extensive tissue destruction and systemic involvement characteristic of electrical trauma.

Overall, the data highlight the need for early recognition, repeated surgical management and close interdisciplinary collaboration in the treatment of HVEI. Standard burn severity scores alone do not adequately capture the clinical complexity of these injuries. Tailored treatment strategies and resource planning are therefore essential to address the unique demands of this patient group.

## Limitations

A limitation of this study is the comparatively small number of patients with high-voltage electrical injuries (HVEI); however, given the rarity of this trauma mechanism, the inclusion of 92 HVEI cases represents 1 of the larger cohorts reported in the international literature, enabling meaningful statistical comparisons with thermally injured patients.
